# Supplementary material for: High Antitumor Activity of the Dual Topoisomerase Inhibitor P8-D6 in Breast Cancer
Source: Cancers (Basel). 2021 Dec 21;14(1):2. doi: 10.3390/cancers14010002 (PMC8750241; doi:10.3390/cancers14010002)
Supplement: Supplementary file 1 [file cancers-14-00002-s001.zip › cancers-1517238-supplementary.pdf]

Supplement Materials: High Antitumor Activity of the Dual Topoisomerase Inhibitor P8-D6 in Breast Cancer

Inken Flörkemeier, Tamara N. Steinhauer, Nina Hedemann, Jörg Paul Weimer, Christoph Rogmans, Marion T. van Mackelenbergh, Nicolai Maass, Bernd Clement and Dirk O. Bauerschlag

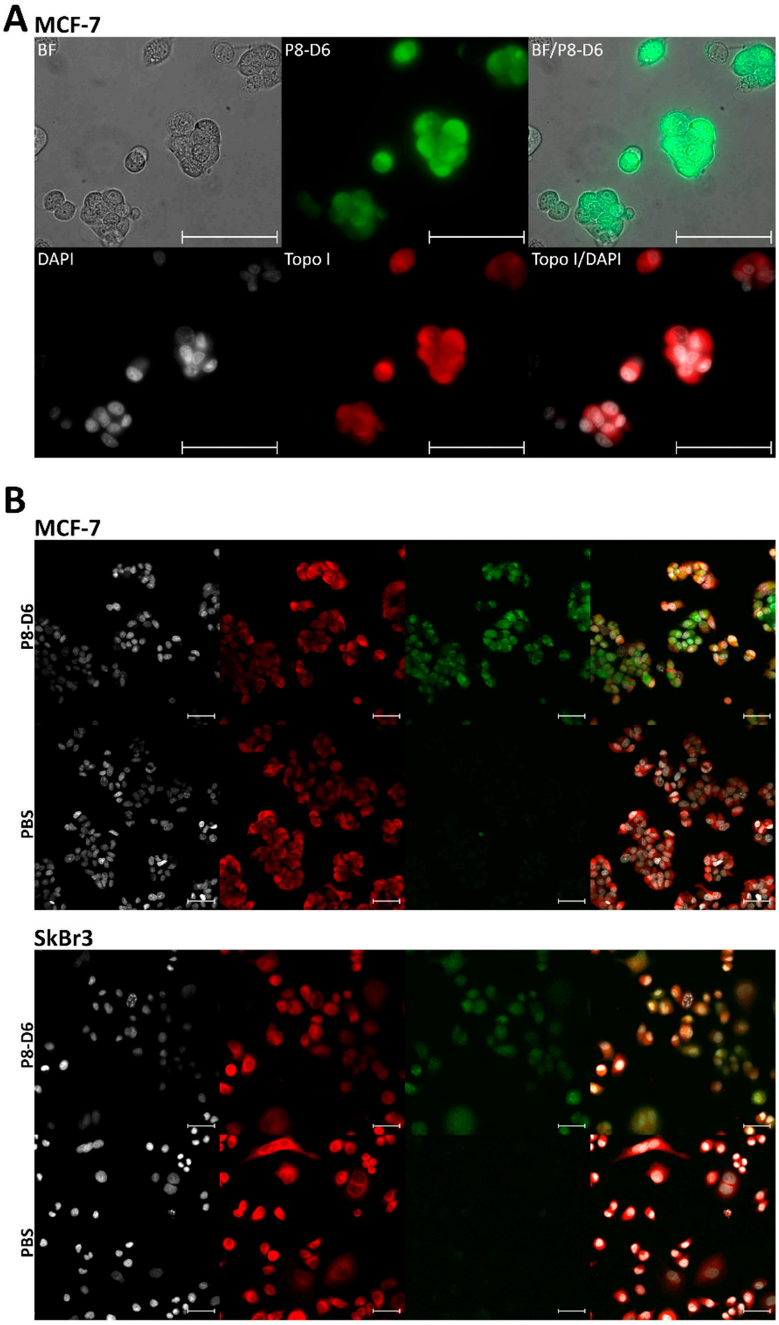

Figure S1. Localization of P8-D6. MCF-7 (**A, B**) and SkBr3 (**C**) cells were treated with 10 µM P8-D6 (fluorophore: 462Ex/530Em) or control (PBS) for 10 h. P8-D6 was localized *in vitro*. (**A**) After fixation topoisomerase I was stained using anti-Topo I antibody (Santa Cruz#sc-271285) and imaged (63x) using LSM 880 and software ZEN 2.5 (blue edition). (**B, C**): After treatment cells were stained with CellTracker™ Deep Red Dye and hoechst 33342 (25x). Fluorescence intensity of P8-D6 was quantified in the nucleus. Fluorescence images show the fluorophore P8-D6 in green, membrane staining (**B, C**) or topoisomerase expression (**A**) in red and nucleus staining in white. Scale bars, 50 µm.

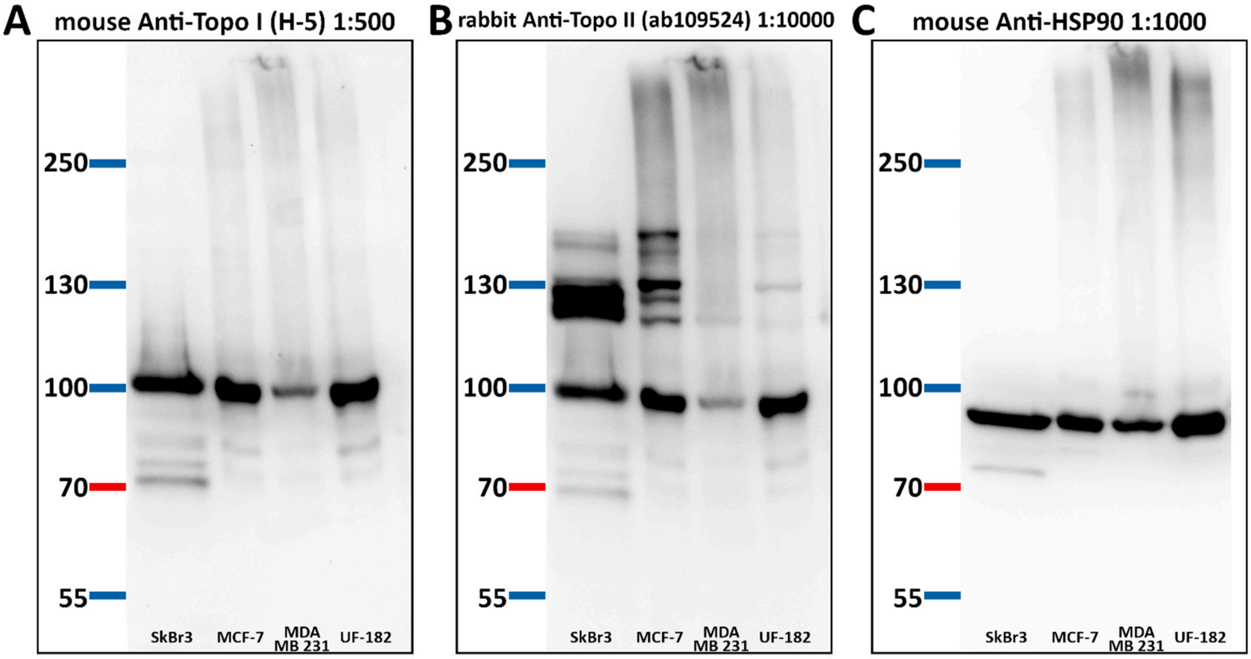

Figure S2. Protein expression of Topoisomerase I/II. Lysates of ovarian cancer cells were analysed by western blot to validate Topo I and Topo II protein expressions. In cell lysates Topo I (**A**) at 100 kDa, Topo II α (**B**) at 174 kDa, Topo II β (**B**) at 180 kDa were detected. HSP 90 (**C**) at 90kDa was used as loading control. After Topo II and before HSP90 detection, the blot was stripped.
